# Supplementary material for: Impact of Rap-Phr system abundance on adaptation of Bacillus subtilis
Source: Commun Biol. 2021 Apr 13;4:468. doi: 10.1038/s42003-021-01983-9 (PMC8044106; doi:10.1038/s42003-021-01983-9)
Supplement: Supplementary file 1 — Supplementary Information [file 42003_2021_1983_MOESM1_ESM.pdf]

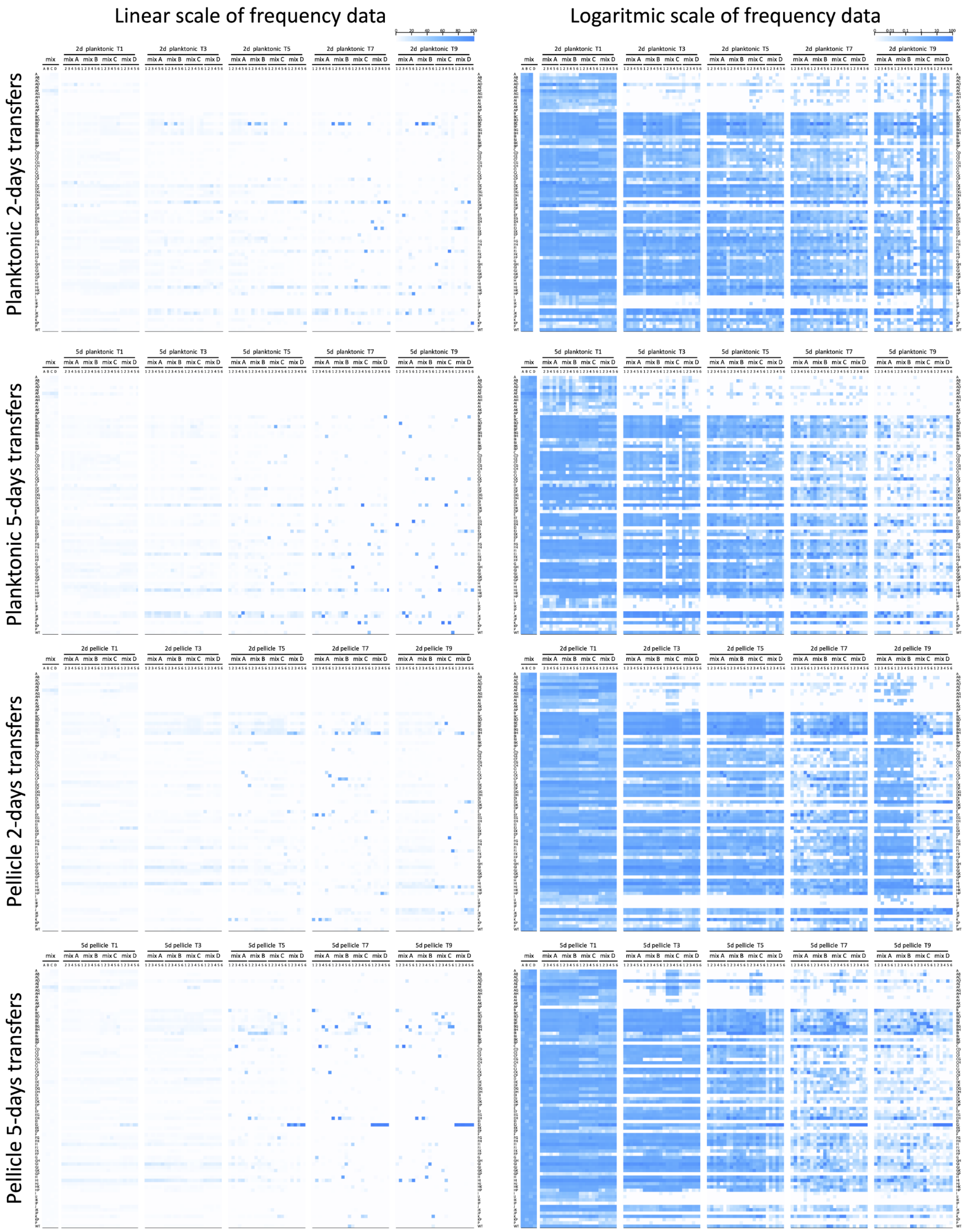

Supplementary Fig. 1 Heat map representation of the population dynamics of *B. subtilis rap-phr* mutants in competition. Boxes represent the population percentage of strains. Text columns at far-left and far-right indicate which *rap-phr* genes have been deleted (A indicates a Δ*rapA* mutant, AB indicates a

Δ*rapA*Δ*rapB* mutant, and so on), WT indicates *B. subtilis* DK1042. Text rows on top indicate type of culture (planktonic or pellicle), incubation period (2d= 2 days, 5d= 5days), transfer number of represented population (t1, t3, t5, t7, and t9), and mix and replicate number. Competition populations were started from 4 population mixes (A to D), with 6 replicates per mix. The first two box columns indicate the population representation of tested strains in the competition starter mixes. Left and right panels show the same data, although using different scales (shown in the top-right corner of each panel): linear increment of percentage (left) and logarithmic increment of percentage (right).

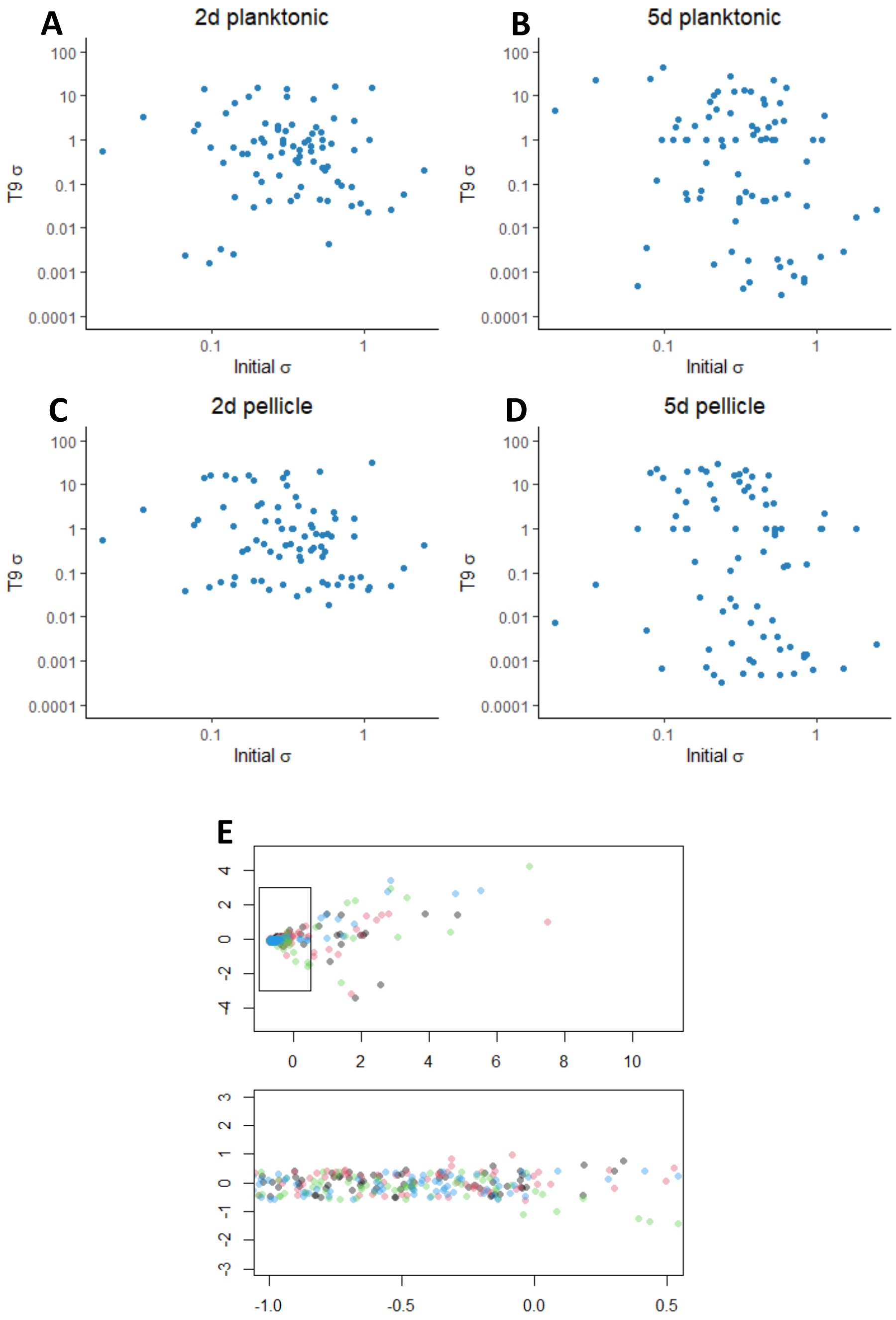

Supplementary Fig. 2 Statistical analysis on the variability between initial mixes and the respective final transfers. (A to D) Standard deviations of relative abundances of each mutant and wild type in the 4 inoculation mixtures (initial σ) versus at the 9th transfers (T9 σ) are spotted on the x- and y-axis, respectively. Panels A, B, C, and D display the plotted data for selection regime 2 days planktonic, 5 days planktonic, 2 days pellicle, and 5 days pellicle, respectively. Panel E depicts a PCA analysis on aggregated and scaled data colored according to the 4 mixes. The most closely clustering observations (marked by the square) were enlarged and shown below for better visualization.

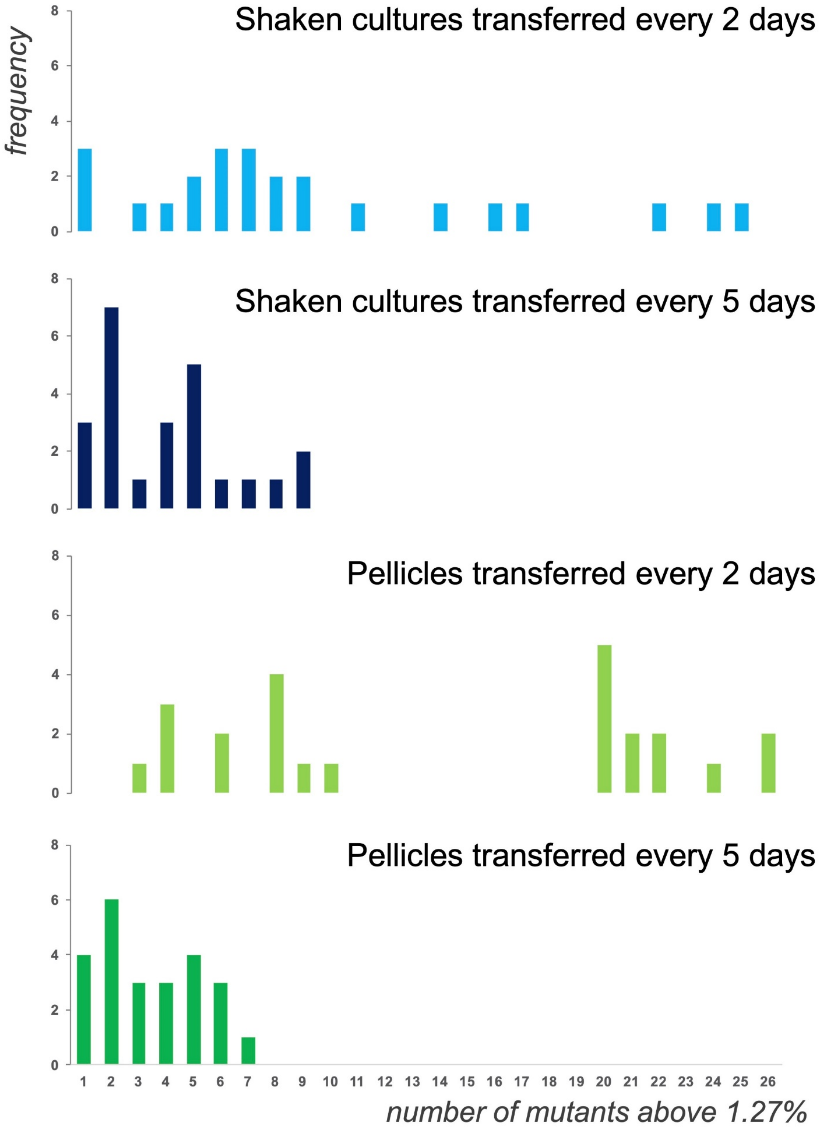

Supplementary Fig. 3 Distribution within the 24 replicates of the number of mutants showing higher relative abundance than the initial 1.27% input, after transfer 9. The X axis of the bar charts represents the number of mutants above 1.27% relative abundance at the end of the competition experiments of the 4 cultivation conditions, while y axis depicts their frequencies within the 24 replicates.

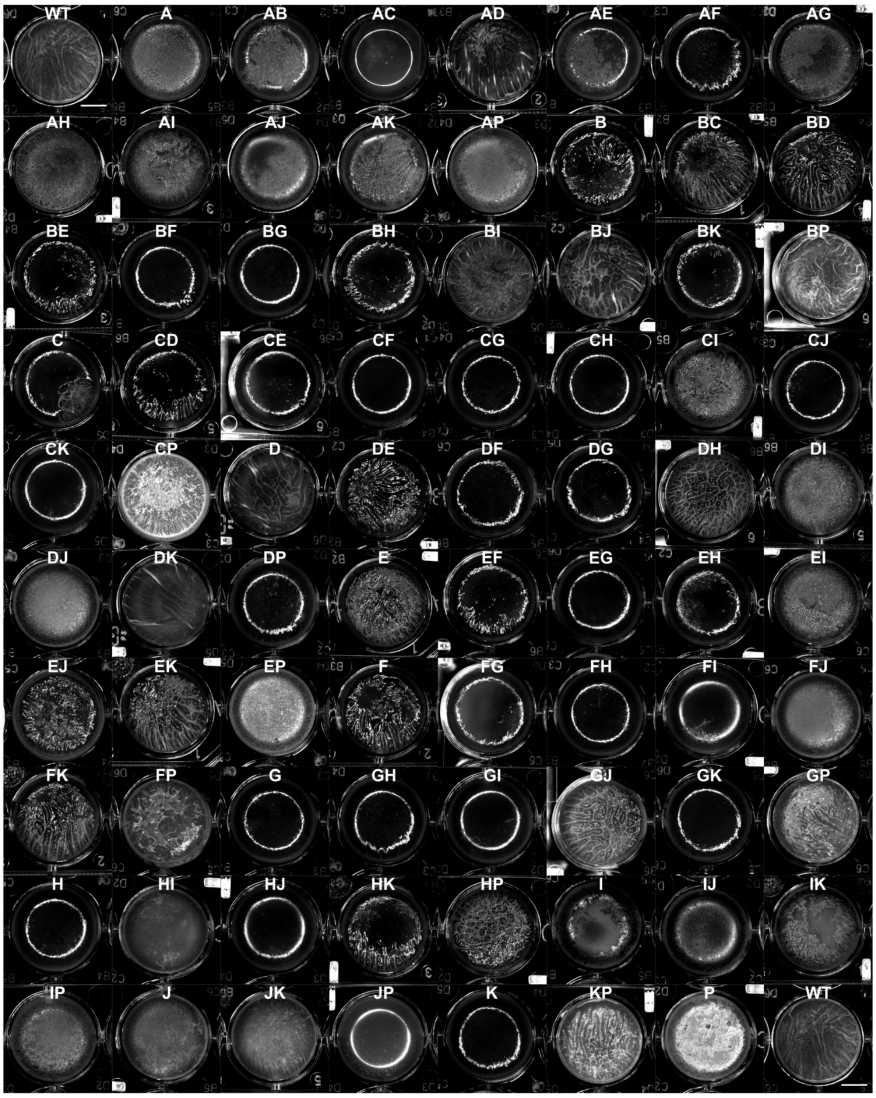

Supplementary Fig. 4. 2 days pellicles of *B. subtilis* DK1042 and *rap-phr* mutants. Bright-field images are shown after 2 days of incubation on MSgg medium at 30°C. WT indicates *B. subtilis* DK1042, A indicates a Δ*rapA* mutant, AB indicates a Δ*rapA*Δ*rapB* mutant, and so on. The images shown here have an adjusted contrast, so that the pellicles can be easily appreciated. The same image of *B. subtilis* DK1042 is presented twice (top-left and bottom-right) to facilitate pellicle comparison. The scale bars represent 5 mm.

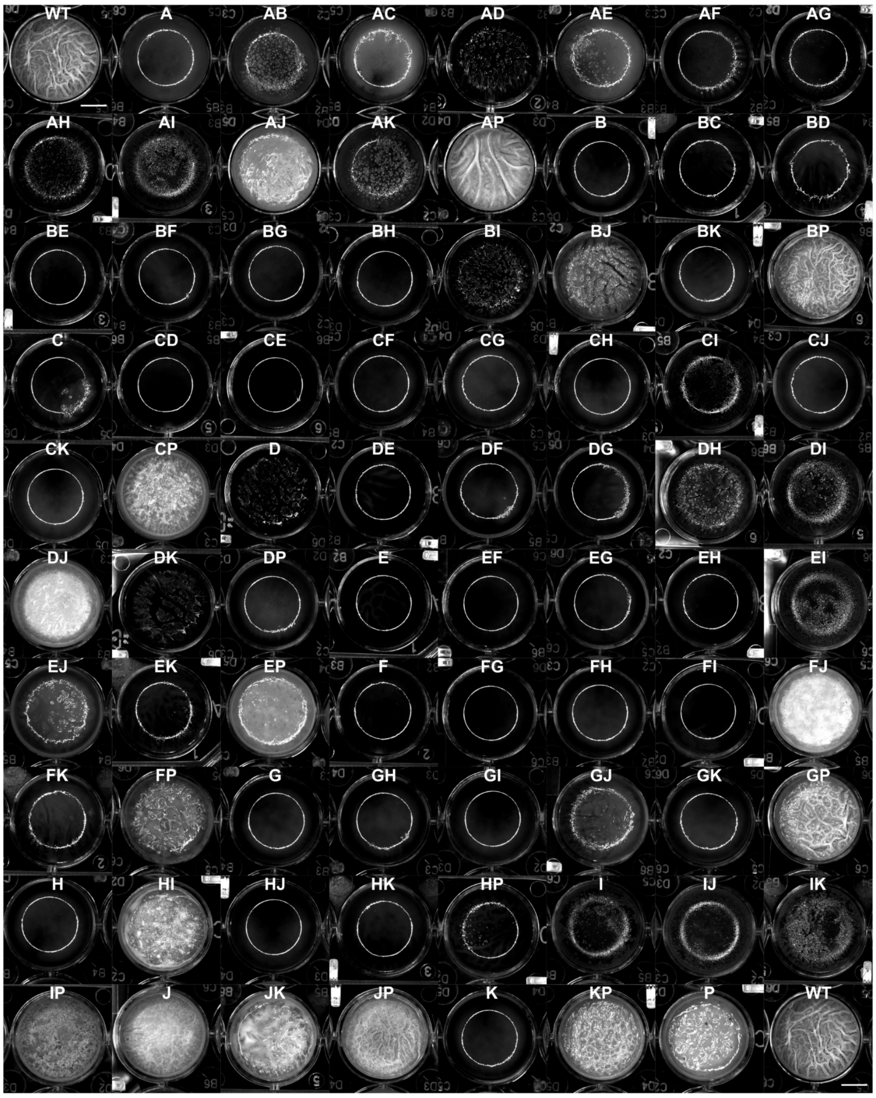

Supplementary Fig. 5. 5 days pellicles of *B. subtilis* DK1042 and *rap-phr* mutants. Bright-field images are shown after 5 days of incubation on MSgg medium at 30°C. WT indicates *B. subtilis* DK1042, A indicates a Δ*rapA* mutant, AB indicates a Δ*rapA*Δ*rapB* mutant, and so on. The images shown here have an adjusted contrast, so that the pellicles can be easily appreciated. The same image of *B. subtilis* DK1042 is presented twice (top-left and bottom-right) to facilitate pellicle comparison. The scale bars represent 5 mm.

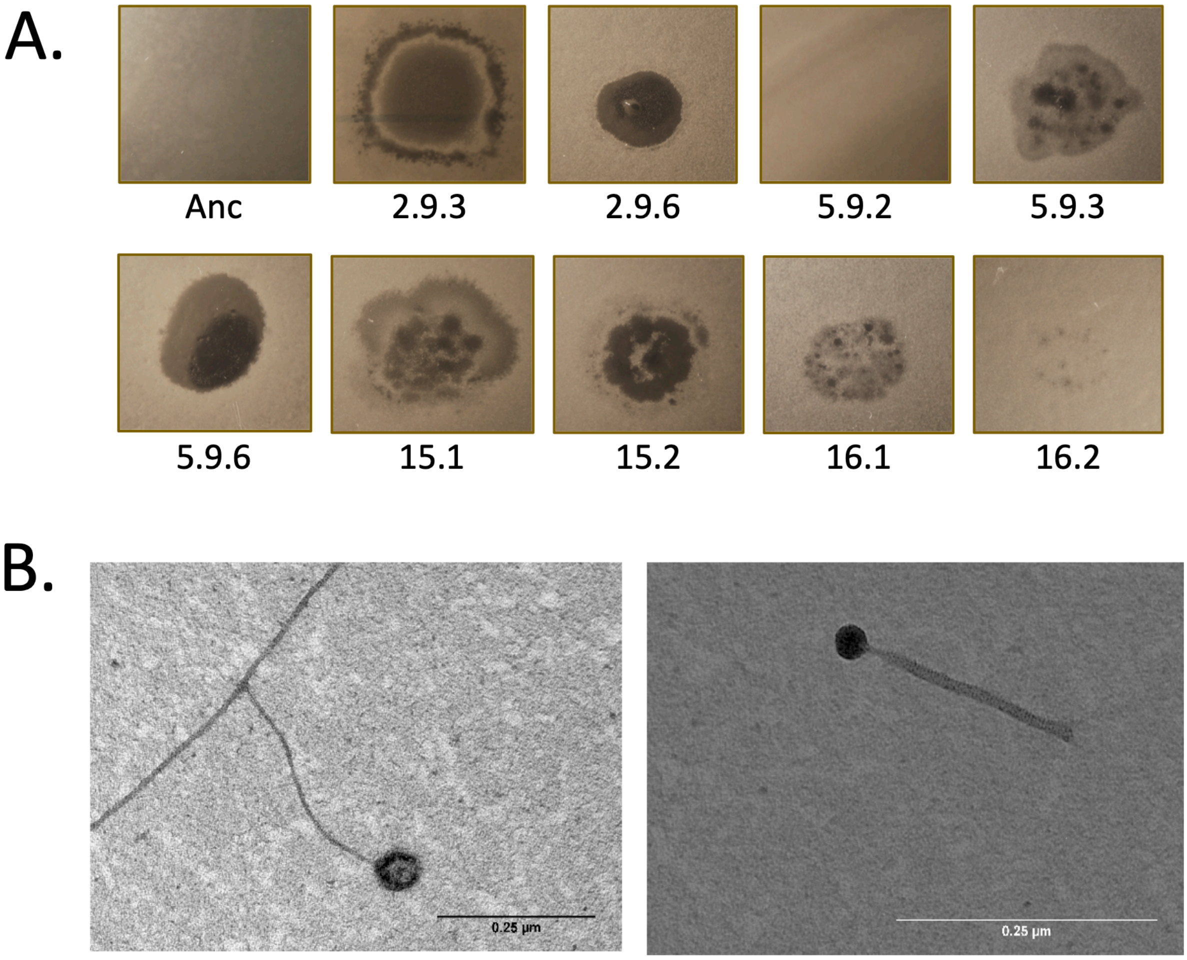

Supplementary Fig. 6. Presence of infective phage particles in the isolated evolved linages. (A) Supernatants, obtained from overnight cultures of the evolved strains, were spotted on soft-agar (0.3%) lawn of the ancestor strain with knockout of biofilm genes (NCBI 3610 Δ*eps*Δ*tasA*). (B) Electron microscopy images of isolated phage particles.

References included in Supplementary Data 2

1. Konkol, M. A., Blair, K. M. & Kearns, D. B. Plasmid-encoded ComI inhibits competence in the ancestral 3610 strain of *Bacillus subtilis*. *J. Bacteriol.* **195**, 4085–4093 (2013).
2. Casadaban, M. J. & Cohen, S. N. Analysis of gene control signals by DNA fusion and cloning in *Escherichia coli*. *J. Mol. Biol.* **138**, 179–207 (1980).
3. Hölscher, T. *et al.* Motility, chemotaxis and aerotaxis contribute to competitiveness during bacterial pellicle biofilm development. *J. Mol. Biol.* **427**, 3695–3708 (2015).
4. Kovács, Á. T., van Hartskamp, M., Kuipers, O. P. & van Kranenburg, R. Genetic tool development for a new host for biotechnology, the thermotolerant bacterium *Bacillus coagulans*. *Appl. Environ. Microbiol.* **76**, 4085–4088 (2010).
5. Grau, R. R. *et al.* A duo of potassium-responsive histidine kinases govern the multicellular destiny of Bacillus subtilis. *MBio* **6**, e00581-15 (2015).
