# Supplementary material for: Impact of Rap-Phr system abundance on adaptation of Bacillus subtilis
Source: Commun Biol. 2021 Apr 13;4:468. doi: 10.1038/s42003-021-01983-9 (PMC8044106; doi:10.1038/s42003-021-01983-9)
Supplement: Supplementary file 7 — Description of Additional Supplementary Files [file 42003_2021_1983_MOESM7_ESM.pdf]

Description of Additional Supplementary Files

File Name: Supplementary Data 1

Description: **List of mutations detected in genomes of sequenced population clones.** Mutations detected in each sequenced clone are indicated in separate tabs, while the last two tabs enlist mutations found in multiple and in single evolved isolates, respectively.

File Name: Supplementary Data 2

Description: Strains and plasmids used in this study.

File Name: Supplementary Data 3

Description: Primers used in this study

File Name: Supplementary Data 4

Description: DNA barcoded strains used in this study.

File Name: Supplementary Data 5

Description: Data for creating Fig 2, Fig 3, Fig 4, Fig 5, and Fig 6
