# Supplementary material for: Impact of Rap-Phr system abundance on adaptation of Bacillus subtilis
Source: Commun Biol. 2021 Apr 13;4:468. doi: 10.1038/s42003-021-01983-9 (PMC8044106; doi:10.1038/s42003-021-01983-9)
Supplement: Supplementary file 9 — Reporting Summary [file 42003_2021_1983_MOESM9_ESM.pdf]

nature research

Corresponding author(s): Akos T Kovacs

Last updated by author(s): Mar 3, 2021

Reporting Summary

Nature Research wishes to improve the reproducibility of the work that we publish. This form provides structure for consistency and transparency in reporting. For further information on Nature Research policies, see our Editorial Policies and the Editorial Policy Checklist.

Statistics

For all statistical analyses, confirm that the following items are present in the figure legend, table legend, main text, or Methods section.

n/a	Confirmed	
☐	☒	The exact sample size (n) for each experimental group/condition, given as a discrete number and unit of measurement
☐	☒	A statement on whether measurements were taken from distinct samples or whether the same sample was measured repeatedly
☐	☒	The statistical test(s) used AND whether they are one- or two-sided<br>*Only common tests should be described solely by name; describe more complex techniques in the Methods section.*
☐	☒	A description of all covariates tested
☐	☒	A description of any assumptions or corrections, such as tests of normality and adjustment for multiple comparisons
☐	☒	A full description of the statistical parameters including central tendency (e.g. means) or other basic estimates (e.g. regression coefficient) AND variation (e.g. standard deviation) or associated estimates of uncertainty (e.g. confidence intervals)
☒	☐	For null hypothesis testing, the test statistic (e.g. F, t, r) with confidence intervals, effect sizes, degrees of freedom and P value noted<br>*Give P values as exact values whenever suitable.*
☒	☐	For Bayesian analysis, information on the choice of priors and Markov chain Monte Carlo settings
☒	☐	For hierarchical and complex designs, identification of the appropriate level for tests and full reporting of outcomes
☒	☐	Estimates of effect sizes (e.g. Cohen's d, Pearson's r), indicating how they were calculated

Our web collection on statistics for biologists contains articles on many of the points above.

Software and code

Policy information about availability of computer code

Data collection: For sequencing, the primary data analysis (base-calling) was carried out with Bbcl2fastq^ software (v2.17.1.14, Illumina), the Software for image acquisition during electron-microscopy was Gatan Microscopy Suite Version 3.21.1374.0, for stereomicroscopy Zen Black 2.0 SR (Zeiss, Germany)

Data analysis: OriginPro 2018b (OriginLab, US), R environment for statistical computing and graphics (The R Foundation), Genesis 1.8.1 (Institute for Genomics and Bioinformatics, Graz University of Technology).

For manuscripts utilizing custom algorithms or software that are central to the research but not yet described in published literature, software must be made available to editors and reviewers. We strongly encourage code deposition in a community repository (e.g. GitHub). See the Nature Research guidelines for submitting code & software for further information.

Data

Policy information about availability of data

All manuscripts must include a data availability statement. This statement should provide the following information, where applicable:

- Accession codes, unique identifiers, or web links for publicly available datasets
- A list of figures that have associated raw data
- A description of any restrictions on data availability

Raw sequencing data has been deposited to NCBI Sequence Read Archive (SRA) database. Bioproject accession number: PRJNA626081

Field-specific reporting

Please select the one below that is the best fit for your research. If you are not sure, read the appropriate sections before making your selection.

☐ Life sciences ☐ Behavioural & social sciences ☒ Ecological, evolutionary & environmental sciences

For a reference copy of the document with all sections, see nature.com/documents/nr-reporting-summary-flat.pdf

Ecological, evolutionary & environmental sciences study design

All studies must disclose on these points even when the disclosure is negative.

Study description	We analyzed the role of a family of phosphatase regulators in Bacillus subtilis, we created single and double mutant strains of these regulators in all possible combinations, and tagged them with a DNA barcode (78 mutants + the wildtype strain). We performed a multi-step sequential competition experiment with all the generated strains to examine the role of the regulators on adaptation to various growth regimes using sporulation as our selective criteria. After each competition step we created a bottleneck by using only spores from that step to inoculate and start th next sequential growth and sporulation cycle. We traced the population dynamics and fitness of all the strains involved in our competition by using Illumina sequencing to detect the prevalence of each strain (by its DNA barcode) during the various growth cycles. At the end of the competition we randomly chose isolates from the experimental populations to resequence their genomes in order to detect possible adapative mutations that may have arisen during the competition, and we compared the fitness of those isolated to the fitness of their cognate ancestors to further examine the impact of any putative adaptive mutation.
Research sample	The research sample consisted of mutant strains of Bacillus subtilis DK1042 that lack one or two (in all possible combinations) Rap-Phr regulatory modules. These mutants and the wildtype strain were mixed in equal ratios at the start of the experimental procedure to represent the putative genetic diversity that may exist in natural environments in regards to these Rap-Phr regulators.
Sampling strategy	At the end of each growth cycle of the experimental competition, spores were obtained from each population replicate and used to inoculate the next iteration of the competition. To do this, a random sample of 1/20th of the entire population was used to select for spores (via heath treatment) and those spores were used to inoculate fresh culture medium in a 1:20 ratio. These sampling and inoculum sizes were selected based on the rationale that the chosen growth regimes would provide enough time (2 or 5 days) to replenish the population before the next selection step and thus avoid population collapse.<br>No statistical tests were used to determine sample sizes. Such was chosen based on experience and knowledge on expected biological variability (which cannot be controlled by the experimenter) and accuracy of the experimental methods used. No data were excluded from the analyses.
Data collection	After the 1st, 3rd, 5th, 7th and 9th competition cycle, Gallegos-Monterrosa R. purified genomic DNA from samples of the studied experimental populations (ensuring that DNA was obtained from both planktonic cells and spores), and used it to PCR amplify the loci containing the DNA barcodes of the competing strains. Bálint B. and Maróti G. then sequenced these PCR amplicons via Illumina sequencing as described in the Methods.<br>For competition experiments between ancestor Δrap-phr mutant and WT, as well as between evolved Δrap-phr mutant and its cognate ancestor, the 1:1 mix competed during pellicle biofilm formation for 2 days (representing one of the four growth regimes applied during the population competition experiment). Data was collected at two time points, the first one being at the start right after inoculation in order to quantify starting CFU of both competitors. The second data collection was after 2 days, where the formed pellicle biofilm was harvested and sonicated. The obtained cell suspension was divided into two: One that was immediately plated for quantification of total CFU, and one which was heat-treated in order to obtain spores only, followed by plating to quantify number of spores.<br>Anna Dragos and Pryadarshini B analyzed the phage activity in single isolates obtained from populations after 9th competition cycle. Isolates were retrieved from -80C, cultivated in LB to obtain supernatants, which were then tested by plaque assay. Identically obtained supernatants were used to purify phage particles for TEM visualization.
Timing and spatial scale	For the populations that were competed under a 2-day growth regime, samples were collected during a 18-day period, accounting for 9 growth cycles. Samples of genomic DNA were obtained from the populations after every 2 growth periods (starting with the first), as well as from the strain mixes used to initiate the experiment.<br>For the populations that were competed under a 5-day growth regime, samples were collected during a 45-day period, accounting for 9 growth cycles. Samples of genomic DNA were obtained from the populations after every 2 growth periods (starting with the first), as well as from the strain mixes used to initiate the experiment.<br>Samples were collected every 2 growth cycles due to budget limitations regarding their sequencing.<br>As stated under "Data collection", the 1:1 competition experiments were competed during pellicle biofilm formation for 2 days, and data collected at the start at end of the 2 days.<br>For plaque assay and TEM analysis, samples were obtained through 16h cultivation of single isolates obtained from populations after 9th competition cycle. Cultivation was performed from -80C stocks inocula, each in triplicate. Plaque assays were performed on fresh supernatants (assay performed at the collection day). Purified phage samples were stored at 4C no longer than 14 days, prior to TEM.
Data exclusions	No data were excluded from the analyses.
Reproducibility	All experiments contained biological replicates, vast majority of the experiments were performed multiple times.<br>To verify the reproducibility of our experimental findings we decided to have multiple replicates of each tested experimental setup (4 growth and selection regimes): we used 24 replicates of each one of these conditions. Furthermore, to avoid the risk of genetic drift biasing our competition results, we initiated these 24 replicates using 4 independent starter strain mixes (6 replicates per mix). These mixes were in turn created using completely independent cultures of each used strain (79 strains in total per mix).

	Lack of any correlation between variability in % of each strain within the inoculation mixes and at the 9th transfer was examined using Spearman's rank correlation.<br>Additionally, to guarantee that our approach was robust and not unduly influenced by environmental conditions (e.g. air humidity), the 24 replicates of each condition were divided in 2 sets of 12 replicates each that were processed completely before starting the competition period of the next set.<br>To verify the experimental findings of the 1:1 competitions, each competition was carried out in 3 to 4 technical replicates (N=3-4).<br>Plaque assay was performed in triplicates for each strain. The assay was reproduced in at least 3 independent experiments (starting from -80C inoculation). Samples for TEM were prepared in duplicates, but the experiment was performed once.
Randomization	Randomization during the collection of samples was guaranteed by keeping the entire population vigorously mixed during the entire growth cycle (planktonic cultures), or by collecting the entire population and sonicating and mixing it immediately prior to sampling (pellicle cultures).<br>Randomization during collection of the samples was taken into account by making sure that the entire population, i.e. the entire pellicle biofilm, was collected and sonicated, and the resulting cell suspension was mixed immediately prior to plating for total CFU and prior to heat treatment (to obtain only spores).<br>Does not apply for plaque assay, as supernatant was collected from the fully grown culture cultivated with shaking at 220 rpm. For TEM, multiple view fields were examined to obtain representative images (<10 images per sample).
Blinding	Blinding was not considered relevant in this study, because methods used in the assessment of the results were objective.

Did the study involve field work? ☐ Yes ☒ No

Reporting for specific materials, systems and methods

We require information from authors about some types of materials, experimental systems and methods used in many studies. Here, indicate whether each material, system or method listed is relevant to your study. If you are not sure if a list item applies to your research, read the appropriate section before selecting a response.

Materials & experimental systems

n/a	Involved in the study
☒	☐ Antibodies
☒	☐ Eukaryotic cell lines
☒	☐ Palaeontology and archaeology
☒	☐ Animals and other organisms
☒	☐ Human research participants
☒	☐ Clinical data
☒	☐ Dual use research of concern

Methods

n/a	Involved in the study
☒	☐ ChIP-seq
☒	☐ Flow cytometry
☒	☐ MRI-based neuroimaging
